# nCOVID-19 Pandemic: From Molecular Pathogenesis to Potential Investigational Therapeutics

**DOI:** 10.3389/fcell.2020.00616

**Published:** 2020-07-10

**Authors:** Md. Tanvir Kabir, Md. Sahab Uddin, Md. Farhad Hossain, Jawaher A. Abdulhakim, Md. Asraful Alam, Ghulam Md Ashraf, Simona G. Bungau, May N. Bin-Jumah, Mohamed M. Abdel-Daim, Lotfi Aleya

**Affiliations:** ^1^Department of Pharmacy, Brac University, Dhaka, Bangladesh; ^2^Department of Pharmacy, Southeast University, Dhaka, Bangladesh; ^3^Pharmakon Neuroscience Research Network, Dhaka, Bangladesh; ^4^Department of Physical Therapy, Graduate School of Inje University, Gimhae, South Korea; ^5^Department of Medical Laboratory, Faculty of Applied Medical Sciences, Taibah University, Yanbu, Saudi Arabia; ^6^School of Chemical Engineering, Zhengzhou University, Zhengzhou, China; ^7^King Fahd Medical Research Center, King Abdulaziz University, Jeddah, Saudi Arabia; ^8^Department of Medical Laboratory Technology, Faculty of Applied Medical Sciences, King Abdulaziz University, Jeddah, Saudi Arabia; ^9^Department of Pharmacy, Faculty of Medicine and Pharmacy, University of Oradea, Oradea, Romania; ^10^Department of Biology, College of Science, Princess Nourah Bint Abdulrahman University, Riyadh, Saudi Arabia; ^11^Department of Zoology, College of Science, King Saud University, Riyadh, Saudi Arabia; ^12^Pharmacology Department, Faculty of Veterinary Medicine, Suez Canal University, Ismailia, Egypt; ^13^Chrono-Environnement Laboratory, UMR CNRS 6249, Bourgogne Franche-Comté University, Besançon, France

**Keywords:** SARS-CoV-2, nCOVID-19, RdRp inhibitors, remdesivir, favipiravir, immunomodulators, corticosteroids, eculizumab

## Abstract

In December 2019, a severe acute respiratory syndrome coronavirus 2 (SARS-CoV-2)-related epidemic was first observed in Wuhan, China. In 2020, owing to the highly infectious and deadly nature of the virus, this widespread novel coronavirus disease 2019 (nCOVID-19) became a worldwide pandemic. Studies have revealed that various environmental factors including temperature, humidity, and air pollution may also affect the transmission pattern of COVID-19. Unfortunately, still, there is no specific drug that has been validated in large-scale studies to treat patients with confirmed nCOVID-19. However, remdesivir, an inhibitor of RNA-dependent RNA polymerase (RdRp), has appeared as an auspicious antiviral drug. Currently, a large-scale study on remdesivir (i.e., 200 mg on first day, then 100 mg once/day) is ongoing to evaluate its clinical efficacy to treat nCOVID-19. Good antiviral activity against SARS-CoV-2 was not observed with the use of lopinavir/ritonavir (LPV/r). Nonetheless, the combination of umifenovir and LPV/r was found to have better antiviral activity. Furthermore, a combination of hydroxychloroquine (i.e., 200 mg 3 times/day) and azithromycin (i.e., 500 mg on first day, then 250 mg/day from day 2–5) also exhibited good activity. Currently, there are also ongoing studies to evaluate the efficacy of teicoplanin and monoclonal and polyclonal antibodies against SARS-CoV-2. Thus, in this article, we have analyzed the genetic diversity and molecular pathogenesis of nCOVID-19. We also present possible therapeutic options for nCOVID-19 patients.

## Introduction

Coronaviruses (CoVs) belong to the large family of positive-sense, enveloped, highly diverse, and single-stranded RNA viruses (Fehr and Perlman, 2015). Indeed, CoVs have been found to infect both humans and animals, therefore causing various respiratory, gastrointestinal, neuronal, and hepatic diseases (Weiss and Leibowitz, 2011; Chan et al., 2013; Zumla et al., 2016). Former epidemics of CoVs include severe acute respiratory syndrome (SARS)-CoV and Middle East respiratory syndrome (MERS)-CoV, these outbreaks caused severe health problems in humans (Raoult et al., 2020). A group of individuals was admitted to hospitals in late December of 2019 with a primary diagnosis of pneumonia due to an unknown cause (Bogoch et al., 2020; Lu et al., 2020). It was assumed by the earlier reports that the onset of a potential CoV epidemic provided the estimation of a reproduction number for the 2019 novel coronavirus (nCOVID-19, named by World Health Organization (WHO) on Feb 11, 2020) which was thought to be considerably >1 (ranges from 2.24–3.58) (Zhao et al., 2020).

This severe acute respiratory syndrome coronavirus 2 (SARS-CoV-2) can be transmitted largely via droplets and due to the close contact. It has been found that elderly people and individuals with chronic diseases or comorbidities are particularly high-risk populations (Li et al., 2020a). There are various symptoms of nCOVID-19 including cough (68%), fever (88%), diarrhea (3.7%), and vomiting (5%) (Mungroo et al., 2020). The mode of transmission of SARSCoV-2 is supposed to take place from human to human through respiratory secretions released by the infected people when sneezing and coughing (Mungroo et al., 2020). nCOVID-19 patients can be asymptomatic, which is making the control of the transmission more difficult (Gao et al., 2020; Li et al., 2020a). Since February of 2020, strict infection control approaches were executed by the Centers for Disease Control (CDC) in order to limit the spread of SARS-CoV-2. In a recent study, To et al. (2020) mentioned that nCOVID-19 patients had the highest viral load (measured in saliva samples) near presentation. They also summarized that as viral load is quite high during the time of hospital admissions, use of potent antiviral agents at an early stage might prove beneficial in managing the severity of nCOVID-19 infection (To et al., 2020).

Previously, SARS was found to be partially linked with environmental factors (Lin et al., 2006). In a study, it was revealed that air pollution was linked with mortality in SARS patients in China (Cui et al., 2003). In this regard, it was mentioned that lung functions can be compromised owing to long- or short-term exposure to certain environmental pollutants (Cui et al., 2003). Air temperature is another factor that is also needed to be considered. It has been revealed by Lin et al. (2006) that the occurrence of SARS was much higher (18 times) at lower air temperatures as compared to higher temperatures. Researchers also showed that respiratory disorders are more likely to take place in colder environments since virulence of agents are likely to deteriorate at higher air temperatures because they might not endure the alterations in the environment (D'Amato et al., 2018). In addition to this, they also summarized that SARS-CoV's transmissibility is comparable with the transmissibility of influenza virus. Moreover, the occurrence of influenza markedly elevates with high relative humidity and low temperatures (Park et al., 2020), which is further suggesting that viral transmission can be significantly affected by environmental factors.

There are no therapeutic agents that have been approved to treat nCOVID-19. Various medicines including immunomodulatory or antiviral drugs such as remdesivir, favipiravir, ribavirin, chloroquine, hydroxychloroquine, azithromycin, nitazoxanide, teicoplanin etc. have been advised as potential investigational drugs, many of which are now being studied in animals and humans (Wang et al., 2020b; WHO, 2020c). On March 28, 2020, the Food and Drug Administration (FDA) gave an emergency use authorization (EUA) for emergency use of oral administrations of chloroquine phosphate and hydroxychloroquine sulfate to treat SARS-CoV-2 infection (FDA, 2020). Along with oxygen and mechanical ventilation, a guideline has also been published by Belgium which involved recommendations from four other European countries, including Switzerland, Netherlands, France, and Italy that recommended the use of remdesivir, lopinavir/ritonavir, tocilizumab, and chloroquine or hydroxychloroquine (Sciensano, 2020). In addition, Japan and China approved the use of favipiravir (an antiviral agent) to treat influenza, which is now under investigation to treat nCOVID-19 (Fujifilm, 2020).

In this article, we have critically appraised the genetic diversity, molecular pathogenesis, symptoms, diagnosis, and prevention of nCOVID-19. Furthermore, we also specially reviewed the mechanisms, efficacy, and use of various drugs that might be beneficial in combating nCOVID-19 infection.

## Genetic Diversity and Evolution of nCOVID-19

In nature, nucleotide substitution is considered as a vital step for viral evolution (Lauring and Andino, 2010). The rapid spreading of SARS-CoV-2 raised a suspicion that mutations are driving its evolution. In a recent study, from GISAID, Phan (2020a) collected 86 complete or near-complete SARS-CoV-2 genomes to estimate its genetic variation. In addition to this, these strains of SARS-CoV-2 were identified in patients with confirmed nCOVID-19 from USA (11), China (50), Japan (5), Australia (5), England (2), Singapore (3), France (4), Germany (1), Belgium (1), South Korea (1), Vietnam (1), and Taiwan (2). ClustalX2 was used to align the pair-wise nucleotide sequence (Saitou and Nei, 1987). As a reference genome, the sequence of the strain “China/WHU01/2020/EPI_ISL_406716” was used. Interestingly, similar to other beta coronaviruses, the genome of SARS-CoV-2 contains a long ORF1ab polyprotein at the 5′ end, followed by 4 main structural proteins, such as nucleocapsid protein, matrix protein, small envelope protein, and spike surface glycoprotein (Phan, 2020b). In addition to this, it was also observed that there were 3 deletions in the genomes of SARS-CoV-2 from Australia (Victoria), USA (Wisconsin), and Japan (Aichi). In contrast, 1 deletion (10 nucleotides) was found in the 3′ end of the genome, while 2 deletions (2 nucleotides and 24 nucleotides) were found in the ORF1ab polyprotein.

Furthermore, it was also observed from the nucleotide sequence alignment that there were 93 missense mutations in the entire genomes of novel coronavirus (Table 1). Except for the envelope protein, 42 mutations were detected in all of the main structural and non-structural proteins. Whereas, 4 missense mutations were observed in the nucleocapsid protein, 1 in the matrix protein, 29 in the ORF1ab polyprotein, and 8 in the spike surface glycoprotein. Interestingly 3 mutations (i.e., Phe^367^, Tyr^364^, and Asp^354^) were found in the spike surface glycoprotein receptor-binding domain. Indeed, spike surface glycoprotein contributes significantly in binding to receptors on the host cell and eventually regulates host tropism (Fung and Liu, 2019). Furthermore, this spike glycoprotein is the main target of neutralizing antibodies (Yu et al., 2020). Conformational changes of spike glycoprotein can be induced by the mutations, which can lead to altered antigenicity. Up until now, no study has identified the amino acids that are involved in conformational alterations of spike glycoprotein. Therefore, further studies are required to identify these important amino acids.

**Table 1:** Several missense mutations have been identified in the entire genome of nCOVID-19 strains (Phan, 2020a).

**Location**	**Number of mutations**	**Codon**	**Mutation**	**Region(s) of strain(s)**
Matrix protein	2		1	
		209	Asp → His	Singapore
3′UTR	3		N/A	
Intergenic region	5		N/A	
Intergenic region	6		N/A	
Nucleocapsid protein	7		4	
		148	Thr → Ile	China (Shenzhen)
		194	Ile → Leu	China (Shenzhen) China (Foshan) USA USA
		202	Ser → Asn	Australia
		344	Pro → Ser	Hong Kong (Guangzhou)
5′ UTR	8		N/A	
Spike polyprotein	14		8	
		32	Phe → Ile	China (Wuhan)
		49	His → Tyr	China (Guangdong)
		247	Ser → Arg	Australia
		354	Asn → Asp	China (Shenzhen)
		364	Asp → Tyr	China (Shenzhen)
		367	Val → Phe	France
		614	Asp → Gly	Germany
		1143	Pro → Leu	Australia
ORF1ab polyprotein	48		29	
		117	Ala → Thr	USA
		309	Pro → Ser	France
		428	Ser → Asn	USA
		609	Thr → Ile	USA
		1176	Ala → Val	Japan
		1599	Leu → Phe	Korea
		1607	Ile → Val	USA
		2194	Met → Thr	China (Shenzhen)
		2235	Leu → Ile	China (Wuhan)
		2244	Ile → Thr	China (Wuhan)
		2251	Gly → Ser	China (Wuhan)
		2345	Ala → Val	China (Shandong)
		2534	Gly → Val	China (Wuhan)
		2579	Asp → Ala	China (Wuhan)
		2708	Asn → Ser	China (Wuhan)
		2908	Phe → Ile	China (Wuhan)
		3058	Thr → Ile	France
		3099	Ser → Leu	China (Shenzhen)
		3606	Leu → Phe	China (Yunnan) China (Shandong) China (Chongqing) Singapore France USA
		3764	Glu → Asp	Japan
		3833	Asn → Lys	China (Wuhan)
		5308	Trp → Cys	Taiwan
		5579	Thr → Ile	USA
		6075	Ile → Thr	England
		6083	Pro → Leu	Japan
		6309	Phe → Tyr	China (Sichuan)
		6565	Glu → Asp	China (Shenzhen)
		6958	Lys → Arg	China (Wuhan)
		7018	Asp → Asn	China (Wuhan)

## Transmission Pattern of nCOVID-19 and Environmental Factors

Various wild and domestic animals such as bats, cats, cattle, and camels might play a role as hosts for coronaviruses (Adhikari et al., 2020). In general, animal coronaviruses do not spread among human beings (CDC, 2020a). Nevertheless, exceptions have been noticed in case of MERS and SARS, where these diseases were found to be transmitted owing to the contact with respiratory droplets from sneezing or coughing of nCOVID-19 patients. Initial nCOVID-19 patients were detected in China, where there was an association with the seafood market of Wuhan, which is indicating that these initial infections took place because of the animal-to-person transmission. Later on, nCOVID-19 was also detected in healthcare professionals and also in other individuals where there was no history of contact with that affected area of Wuhan, which is further suggesting the human-to-human transmission (Gralinski and Menachery, 2020; Huang et al., 2020; Li et al., 2020b; Liu et al., 2020; WHO, 2020d).

As per the recent guidelines from health authorities of China (Adhikari et al., 2020; WHO, 2020e), there are 3 major routes of nCOVID-19 transmission including droplets transmission, aerosol transmission, and contact transmission. Transmissions via droplets were found to take place when respiratory droplets of infected individuals are inhaled or ingested by people who are in close contact. Whereas, contact transmission might take place when a person touches a virus-contaminated-object or surface and then that person touches his/her nose, mouth, or eyes. On the other hand, aerosol transmission might take place when respiratory droplets mix into the air, thus forms aerosols and might result in infection when a high dose of aerosols are inhaled into the lungs in a comparatively closed environment (Adhikari et al., 2020; WHO, 2020e). In a study, it was revealed that the digestive system is also a possible route for SARS-CoV-2 transmission. Symptoms like diarrhea and abdominal discomfort have been observed in individuals with confirmed nCOVID-19, this observation led to studies which revealed that ACE2 (to which SARS-CoV-2 binds) is highly expressed in enterocytes of colon and ileum (Zhang et al., 2020b).

### Temperature

The effect of temperature on the health of humans can be varied depending on the countries or even areas (Hajat and Kosatky, 2010). In line with this aforesaid finding, it was also reported that temperature can affect the transmission of respiratory syndromes-causing viruses including SARS-CoV-2 (Ma et al., 2020) and influenza virus (Park et al., 2020). Studies have also revealed that novel coronavirus and influenza virus can survive only in some specific environmental conditions and their transmissions also depend on temperatures (Chan et al., 2011; Jaakkola et al., 2014), which is also applicable for SARS-CoV-2 transmission (Wang et al., 2020c). It was observed in case of influenza virus that it can transmit more readily at lower temperatures (Lowen and Steel, 2014), since host immunity is likely to remain weakened in cold weather, this can further increase the vulnerability toward infection (Kudo et al., 2019). As the transmission process of coronaviruses is comparable with the influenza virus transmission (Lin et al., 2006), thus it can be expected that these processes are also applicable for the SARS-CoV-2 transmission (Wang et al., 2020c).

### Other Environmental Factors

Several other environmental factors can affect the link between mortality and temperature including air pollution (Cai et al., 2007), humidity (Jaakkola et al., 2014; Kudo et al., 2019), latitude (Bao et al., 2016). In this regard, socio-demographic factors including income, age, and gender (Bao et al., 2016) have also been reported to play roles. In a study, Chan et al. (2011) revealed that individuals who live at lower latitudes showed a strong adaptive capacity toward heat, and a relatively weak adaptive capacity was observed toward cold. These researchers also observed that the viability of SARS-CoV was much lower at higher relative humidity and higher temperatures (for example, relative humidity: over 95%, and temperature: 38°C). In a different study, it was revealed that humidity and temperature are linked with an increased risk of nCOVID-19 (Wang et al., 2020c). Interestingly, coronaviruses can persist on inanimate surfaces including plastic, glass, or metal for up to 9 days (Kampf et al., 2020).

## Molecular Pathogenesis Underlying nCOVID-19

**Figure 1:**
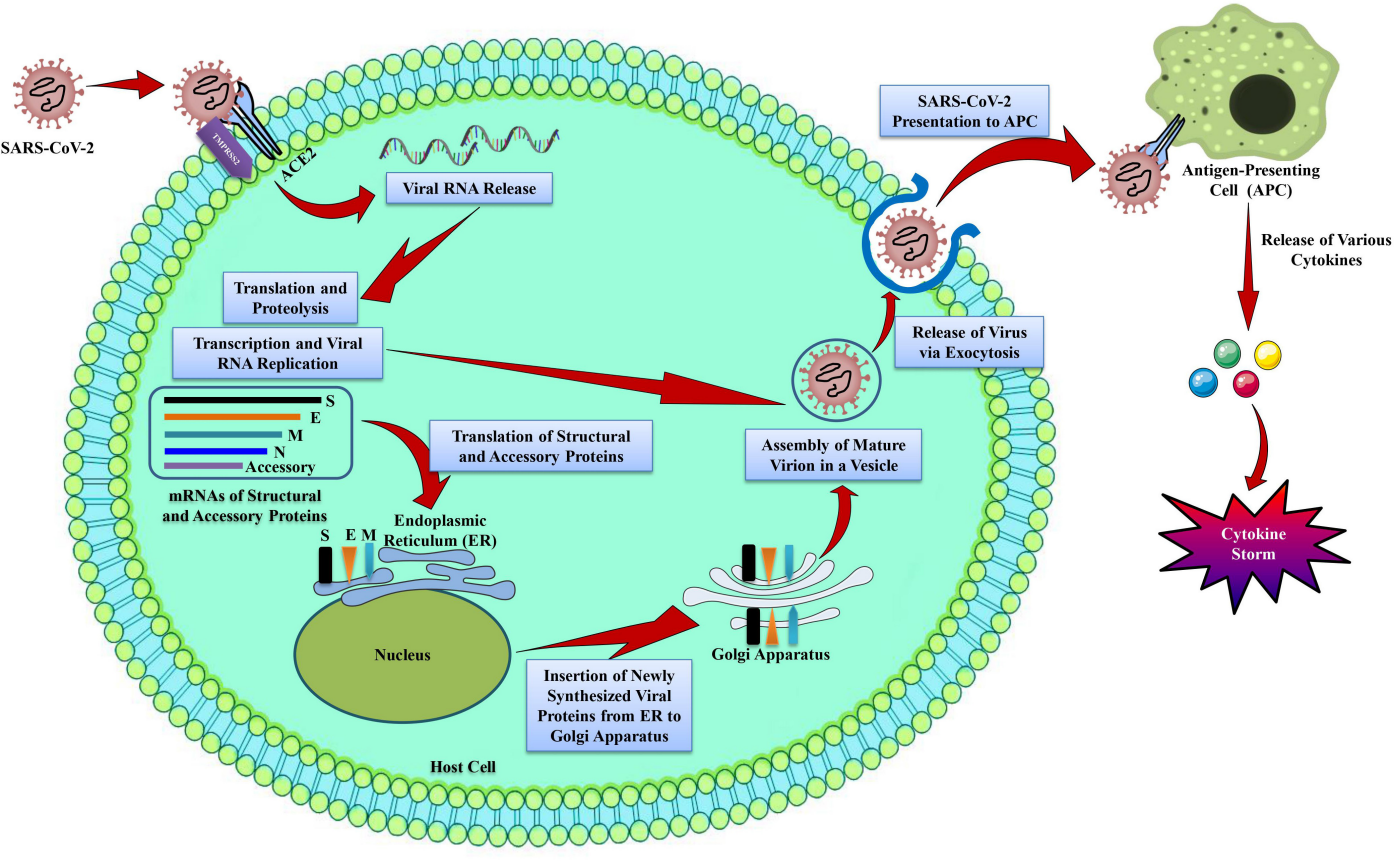
The life cycle of SARS-CoV-2 in host cells. SARS-CoV-2 contains 4 structural proteins including spike (S), envelope (E), matrix/membrane (M), and nucleocapsid (N), in association with various accessory proteins. SARS-CoV-2 enters into the host cell by binding with the S protein of the virus to the ACE2 receptor on the host cell. It has been found that S protein is cleaved into S1 and S2 by a cell-derived protease, where S1 binds with ACE2 receptor, and S2 is activated by the host serine protease TMPRSS2 and results in a fusion with the cell membrane. Following the entry into the host cell, SARS-CoV-2 takeovers the host cell machinery to transcribe, replicate, and translate its RNA genome and structural proteins before being reassembled, encapsulated, and exocytosed from the host cell. Following exocytosis, SARS-CoV-2 is presented to host antigen presenting cells (APCs), which eventually leads to the generation of various cytokines including, TNF-α, CXCL-10, IL-1, and IL-6 (InvivoGen, 2020).

nCOVID-19 patients exhibit various clinical symptoms including cough, fever, fatigue, radiographic evidence of pneumonia, dyspnea, decreased or normal leukocyte counts, and myalgia (Huang et al., 2020). These aforesaid symptoms are also similar to MERS-CoV and SARS-CoV infections (Peiris et al., 2004). Even though nCOVID-19 pathogenesis is not well-understood, however the similar mechanisms used previously by MERS-CoV and SARS-CoV can provide a lot of information regarding SARS-CoV-2 pathogenesis (Figure 1).

### Entry and Replication

Spike protein (S protein) of coronavirus determines the viral entry into the host cells (de Wit et al., 2016). Interestingly, the envelope spike glycoprotein binds to its cellular receptor, angiotensin converting enzyme 2 (ACE2) for SARS-CoV (Li et al., 2003b) and SARS-CoV-2 (Wu et al., 2020b) (Figure 1), dipeptidyl peptidase 4 for MERS-CoV (Raj et al., 2013), and CD209L for SARS-CoV [26]. Although it was initially identified that SARS-CoV enters into cells by direct fusion of plasma membrane and virus (Simmons et al., 2004). However, Belouzard et al. (2009) revealed that a vital proteolytic cleavage process takes place at SARS-CoV S protein at position (S2′) that facilitated the membrane fusion and infectivity of the virus. For membrane fusion, MERS-CoV also has evolved an aberrant 2 steps furin activation (Mille and Whittaker, 2014). Other than membrane fusion, entry of SARS-CoV was also found to be mediated by the clathrin-independent and -dependent endocytosis (Wang et al., 2008; Kuba et al., 2010). Following the entry of virus into the cells, RNA genome of SARS-CoV is released into the cytoplasm and is translated into 2 polyproteins and structural proteins, subsequently the viral genome starts to replicate (Perlman and Netland, 2009). The newly generated envelope glycoproteins are then inserted into the membrane of the endoplasmic reticulum or Golgi apparatus, and the nucleocapsid is generated by the combination of nucleocapsid protein and genomic RNA. Subsequently, viral particles begin to germinate into the endoplasmic reticulum-Golgi intermediate compartment. Finally, virus particles containing vesicles then form fusion with the plasma membrane in order to release the virus (de Wit et al., 2016).

### Antigen Presentation in SARS-CoV-2 Infection

When the SARS-CoV-2 enters into the cells, its antigen will be presented to the antigen presentation cells (APCs) (Figure 1), this process is crucial for the anti-viral immunity of the human body (Kumar et al., 2020). Peptides of antigens are presented via major histocompatibility complex (MHC; or human leukocyte antigen (HLA) in humans) and then identified by virus-specific cytotoxic T lymphocytes. Therefore, the understanding of antigen presentation (AP) of the virus will provide a better understanding of the pathogenesis of nCOVID-19. However, not much information is available regarding this, thus we can obtain information from previous studies on MERS-CoV and SARS-CoV. AP of SARS-CoV-2 mostly relies on MHC I molecules (Liu et al., 2010), nonetheless MHC II also plays roles in its presentation. Former studies revealed that many HLA polymorphisms associate with the susceptibility of SARS-CoV, for instance HLA-Cw*0801 (Chen et al., 2006b), HLA-B*0703, HLA-DR B1*1202, and HLA-B*4601 (Keicho et al., 2009), while HLA-A*0201, HLA-DR0301, and HLA-Cw1502 alleles are associated with the protection from SARS infection (Wang et al., 2011). In case of MERS-CoV, it was observed that MHC II molecules (for example HLA-DQB1*02:0 and HLA-DRB1*11:01) were linked with the susceptibility to MERS-CoV infection (Hajeer et al., 2016). Other than mannose-binding lectin gene polymorphisms linked with AP are associated with the risk of SARS-CoV infection (Tu et al., 2015). Indeed, the aforementioned findings will give us an important idea regarding the mechanism, prevention, and treatment of nCOVID-19.

### Humoral and Cellular Immune Responses

AP subsequently induces the human body's humoral and cellular immune responses, which are then facilitated via virus-specific B and T cells. Like other common acute viral infections, antibodies including IgG and IgM are produced against SARS-CoV virus. It is estimated that at the end of week 12, SARS-specific IgM antibodies disappear. Whereas, SARS-specific IgG antibody can stay for a longer period, which is suggesting that IgG mainly has a protective function (Li et al., 2003a). Furthermore, it was also found that SARS-specific IgG antibodies mainly are N-specific and S-specific antibodies (de Wit et al., 2016). Most of the studies have focused on cellular immune responses, as compared to the humoral immune responses in case of coronavirus. Recent findings have revealed that the levels of CD8^+^ and CD4^+^ T cells in the peripheral blood of nCOVID-19 individuals were significantly decreased, as confirmed by increased percentages of CD38 (CD8 39.4%) and HLA-DR (CD4 3.47%) double-positive fractions (Xu et al., 2020). Likewise, acute phase response in individuals with nCOVID-19 is linked with a marked decrease of CD8^+^ T and CD4^+^ T cells. Interestingly, it was found that although there is no presence of antigen, CD8^+^, and CD4^+^ memory T cells can last for 4 years in individuals who have recovered from SARS-CoV and can perform IFN-γ generation, delayed-type hypersensitivity response and T cell proliferation (Fan et al., 2009). After 6 years of infection with SARS-CoV, specific T-cell memory responses to the SARS-CoV S peptide library can still be identified in 14 of 23 recovered SARS individuals (Tang et al., 2011). In mouse models, specific CD8^+^ T cells also exhibited similar activity in the clearance of MERS-CoV (Zhao et al., 2014). Indeed, these results might be useful in the rational designing of an effective vaccine against SARS-CoV-2.

### Cytokine Storm in nCOVID-19

Acute respiratory distress syndrome (ARDS) is considered as the major cause of nCOVID-19-related death. In the early stages of the epidemic, 6 out of the 41 admitted patients with confirmed nCOVID-19 died owing to ARDS (Huang et al., 2020). This ARDS is found to be the main immunopathological characteristic of SARS-CoV, MERS-CoV, and SARS-CoV-2 infections (Xu et al., 2020). Cytokine storm is the major characteristic of ARDS. This storm is a fatal uncontrolled systemic inflammatory response that takes place because of the high secretions of chemokines (i.e., C-X-C motif chemokine 10 [CXCL10], CXCL9, CXCL8, C-C motif chemokine ligand 5 [CCL5], CCL3, CCL2, etc.) and pro-inflammatory cytokines (i.e., transforming growth factor-β [TGFβ], tumor necrosis factor alpha [TNFα], interleukin 33 [IL-33], IL-18, IL-12, interferon gamma [IFNγ], IL-6, IL-1β, IFN-α, etc.) via immune effector cells in case of SARS-CoV infection (Cameron et al., 2008; Williams and Chambers, 2014; Channappanavar and Perlman, 2017; Huang et al., 2020) as shown in Figure 1. Like SARS-CoV, MERS patients showed increased levels of CXCL-10, CXCL8, CCL5, IFN-α, and interleukin 6 (IL-6) in serum as compared to individuals with the mild to moderate disease (Min et al., 2016). In the human body, a powerful cytokine storm will induce an aggressive attack by the immune system, which will lead to multiple organ failure and ARDS, and will ultimately result in death in severe novel coronavirus infection, as like MERS-CoV and SARS-CoV infection (Xu et al., 2020).

### Immune Evasion by SARS-CoV-2

Various strategies are used by viruses including SARS-CoV and MERS-CoV to evade immune responses for their better survival in host cells. The pattern recognition receptors (PRRs) can identify the evolutionarily conserved microbial structures called pathogen-associated molecular patterns. Nonetheless, MERS-CoV and SARS-CoV can stimulate the generation of double-membrane vesicles lacking PRRs and subsequently can replicate in these vesicles, thus evading the host detection of their double-strand RNA (dsRNA) (Snijder et al., 2006). IFN-I (IFN-β and IFN-α) plays a protective function on MERS-CoV and SARS-CoV infection, however the IFN-I mechanism is suppressed in infected mouse models (Channappanavar et al., 2016, 2019). Interestingly, by directly interacting with the dsRNA, MERS-CoV's accessory protein 4a might block the stimulation of IFN at the level of melanoma differentiation-associated protein 5 activation (Niemeyer et al., 2013). IFN β promoter activation and transportation of IFN regulatory factor 3 to the nucleus can be inhibited by the ORF5, ORF4b, ORF4a, and membrane proteins of MERS-CoV (Yang et al., 2013). SARS-CoV-2 can also affect the AP. In this regard, for instance, gene expression associated with AP is downregulated following MERS-CoV infection (Menachery et al., 2018). Therefore, it is vital to terminate the immune evasion of coronavirus to develop specific and effective therapies.

## Clinical Manifestations of nCOVID-19 Patients

**Figure 2:**
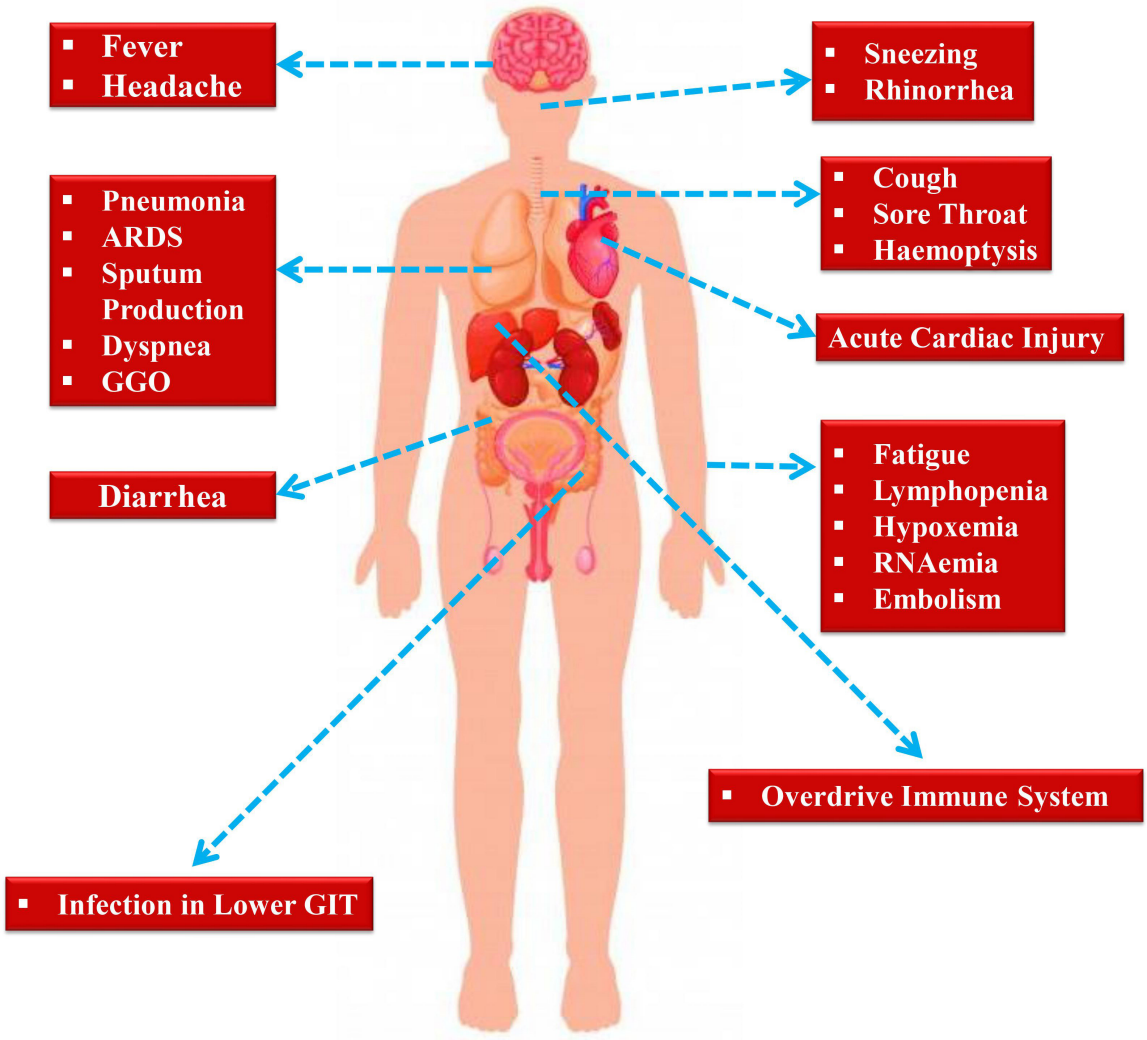
Symptoms exhibited by nCOVID-19 patients.

Following an incubation period of around 5.2 days, the symptoms of SARS-CoV-2 infection appear (Li et al., 2020b). It takes around 6 to 41 days from the first appearance of the symptoms to death, along with a median of 14 days (Wang et al., 2020d). However, the aforesaid durations depend on various factors including the patient's age and status of the immune system. This duration was found to be shorter for individuals older than 70-years old as compared to the individuals who are under the age of 70 (Wang et al., 2020d). At the onset of the disease, the most commonly observed symptoms are cough, fatigue, and fever (Figure 2). In addition to this, various other symptoms including headache, lymphopenia, dyspnea, sputum production, diarrhea, and hemoptysis (Graham Carlos et al., 2020; Huang et al., 2020; Ren et al., 2020; Wang et al., 2020d). Pneumonia has also been identified by computed tomography scan in nCOVID-19 patients, Unfortunately, various aberrant clinical features including ground-glass opacity (GGO), acute cardiac injury, and ARDS led to death (Huang et al., 2020). Occasionally, in subpleural areas of both lungs, the multiple peripheral GGOs were detected (Lei et al., 2020) and these triggered both localized and systemic immune responses, which collectively raised the level of inflammation. Unfortunately, treatment with interferon inhalation did not result in any clinical benefit, rather it aggravated the condition via facilitating pulmonary opacities (Lei et al., 2020).

Indeed, some of the symptoms of nCOVID-19 are similar to the earlier betacoronavirus including dyspnea, dry cough, fever, and bilateral GGOs (Huang et al., 2020). Nonetheless, there are some unique clinical manifestations of nCOVID-19 such as sore throat, sneezing, rhinorrhea (Lee et al., 2003; Assiri et al., 2013). As revealed by chest radiographs following admission, in some cases it was observed that an infiltrate in lung's upper lobe is linked with growing dyspnea with hypoxemia (Phan et al., 2020). Although nCOVID-19 exhibited digestive disorders like diarrhea, only a small proportion of SARS-CoV or MERS-CoV showed similar gastrointestinal symptoms. Thus, testing urine and fecal samples are important to eliminate a possible alternative mode of transmission (Lee et al., 2003; Assiri et al., 2013). Henceforth, developing methods to detect different routes of transmission for example urine and fecal samples are immediately required to develop ways to suppress and/or minimize the transmission and also to discover therapies to treat nCOVID-19.

Recently, it has been observed that nCOVID-19 might predispose to both arterial and venous thromboembolic disease because of immobilization, hypoxia, inflammation, and diffuse intravascular coagulation (Chen et al., 2020b; Guan et al., 2020; Klok et al., 2020; Wang et al., 2020a; Zhou et al., 2020). Furthermore, it was also revealed that respiratory failure in the disease is not only driven by the ARDS, rather microvascular thrombotic activities might also contribute in this regard (Grillet et al., 2020). Therefore, Klok et al. (2020) have strongly suggested to administer pharmacological agents in a prophylactic manner to all the intensive care unit (ICU) nCOVID-19 patients.

## Diagnosis of nCOVID-19 Patients

For any given emergency department (ED) visiting patients with the symptoms of fever and respiratory diseases, healthcare workers must need to get a travel history in detail from that patient. If a patient shows flu-like symptoms and has a travel history to a country or area with confirmed nCOVID-19 cases or if the patient came into close contact with a confirmed nCOVID-19 patient in the last 14 days then the patient ought to be considered as a patient under investigation (PUI) (NPR, 2020). It needs to be noted that here close contact means any individual who was within six feet of an individual with confirmed nCOVID-19 for an extended period. Furthermore, any individual who came into direct contact with the secretions of any nCOVID-19 patient will also be considered as a close contact.

Individuals who have traveled from high-risk countries or areas with confirmed nCOVID-19 cases and members of a family who are suffering from nCOVID-19 and not staying at home care or not maintaining isolation precautions are regarded as high-risk exposures. While medium risk exposures involve individuals who have traveled from low-risk countries or areas and family members are stringently maintaining appropriate home care and adhering with proper isolation precautions (WHO, 2020e). In contrast, low-risk exposures involve those individuals who were in the same indoor environment (for example in a waiting hall) for a longer period with nCOVID-19 patients but did not come into close contact.

Molecular assays of respiratory specimens are performed for diagnosis purposes usually at the regional referral laboratories designated by WHO (Kaiser Health News, 2020). For regional testing, the CDC started distributing nCOVID-19 test kits on February 7 (WHO, 2020a). nCOVID-19 test is getting more widely available day by day. For hospitals or institutions where nCOVID-19 test is not available, the only option is the testing by CDC. nCOVID-19 should be tested on an urgent basis for the PUI cases. An individual should be removed from PUI status only if that individual is fully evaluated clinically and has consulted with proper healthcare professionals.

## Key Messages and Measures for nCOVID-19 Prevention

The mode of SARS-CoV-2 transmission is still complex. Guidelines for nCOVID-19 prevention is mainly based on the previously developed guidelines for SARS and MERS and also on the intervening guidelines provided by CDC and WHO (CDC, 2020a,b; WHO, 2020a). Before or upon arrival in ED, a PUI ought to be identified by the hospitals to protect the healthcare professionals and other patients. Prevention measures should involve maintaining hand and respiratory hygiene and also screening questions including travel history. Following a PUI identification, both local health department and hospital infection control ought to be immediately notified to avert further spread among healthcare professionals and other patients. A surgical mask must need to be given to any PUI and need to be isolated in a private room or if possible in a negative pressure room (WHO, 2020a).

As like SARS and MERS, nCOVID-19 is also found to spread through the airborne route. Therefore, surgical face masks might be beneficial to prevent sneeze and cough-related larger fluid droplets, however they are less likely to prevent small airborne contaminants (Yee et al., 2020). In this regard, respirators containing air filters and adequate seal should be more beneficial (Tran et al., 2012; Smith et al., 2016). In healthcare settings, right use of respirators and personal protective equipment and proper hand hygiene are likely to prevent transmission (Cowling et al., 2009; Radonovich et al., 2019; Yee et al., 2020). If a patient requires hospital admission and there is no private or separate room for that patient, then that patient needs to be taken to an adequate facility containing institution. Isolated rooms and care provide would need to be customized in a way that reduces the exposure of healthcare providers to the patient. Indeed, along with an eye shield, all the healthcare providers must take measures to prevent contact with droplets and to maintain airborne precautions. Since the risk of transmission is much higher during the aerosol-generating procedures (such as intubation), in these cases the importance of PPE is enormous (Raboud et al., 2010; CDC, 2020a). Still, it remains not known, regarding how long nCOVID-19 can stay airborne following a patient leaves the room. Respiratory protection is essential to enter into the vacated room.

### Effectiveness of Personal-Level Prevention

Since still there is no specific drug to treat nCOVID-19, therefore the best approach will be taking preventative measures at a personal level including avoiding public transport, unnecessary travel, contact with nCOVID-19 suspected individuals, and so on.

#### Importance of Hand Washing

Indeed, the significance of maintaining frequent and proper hand hygiene is paramount. Like other coronaviruses, SARS-CoV-2 has a lipid envelope, thus proper hand-washing with soap can break apart that lipid envelope and therefore can make it difficult or even impossible for the virus to infect humans. So far, this proper hand-washing is considered as the most effective preventative measure. In addition, duration of hand-washing with soap is also equally important. CDC has recommended that effective hand-washing should last at least for 20 s. In a study, Borchgrevink et al. (2013) showed that out of 3,749 individuals in a college town environment, only 5% of those individuals properly followed the hand-washing rules (i.e., washing, rubbing, and rinsing). This finding indicates that there is a poor understanding of the significance of proper hand-washing among the general people. Therefore, awareness among people should be increased about the importance of frequent and proper hand-washing.

#### Proper Use of Face Mask

In order to form a physical barrier, the WHO has recommended the use of a face mask by those individuals who are showing respiratory symptoms (WHO, 2020b). However, healthy people are not required to use face masks. A typical surgical mask only provides one-way protection and can avert the spreading of droplets during coughing and sneezing to the surrounding areas. Healthcare professionals who are treating or in contact with a suspected or confirmed nCOVID-19 patient must need to wear a specialized respirator (for example N95 or its equivalent) to effectively prevent the droplets entry and thus can reduce the chance of acquiring the infection (Bae et al., 2020; WHO, 2020b). Strict precautionary measures must need to be taken by the individuals during handling affected individual's body secretions including sputum, urine, or stools (Yeo et al., 2020).

## Therapeutic Options for nCOVID-19 Patients

### Inhibitors of RNA-Dependent RNA Polymerase

#### Remdesivir

**Figure 3:**
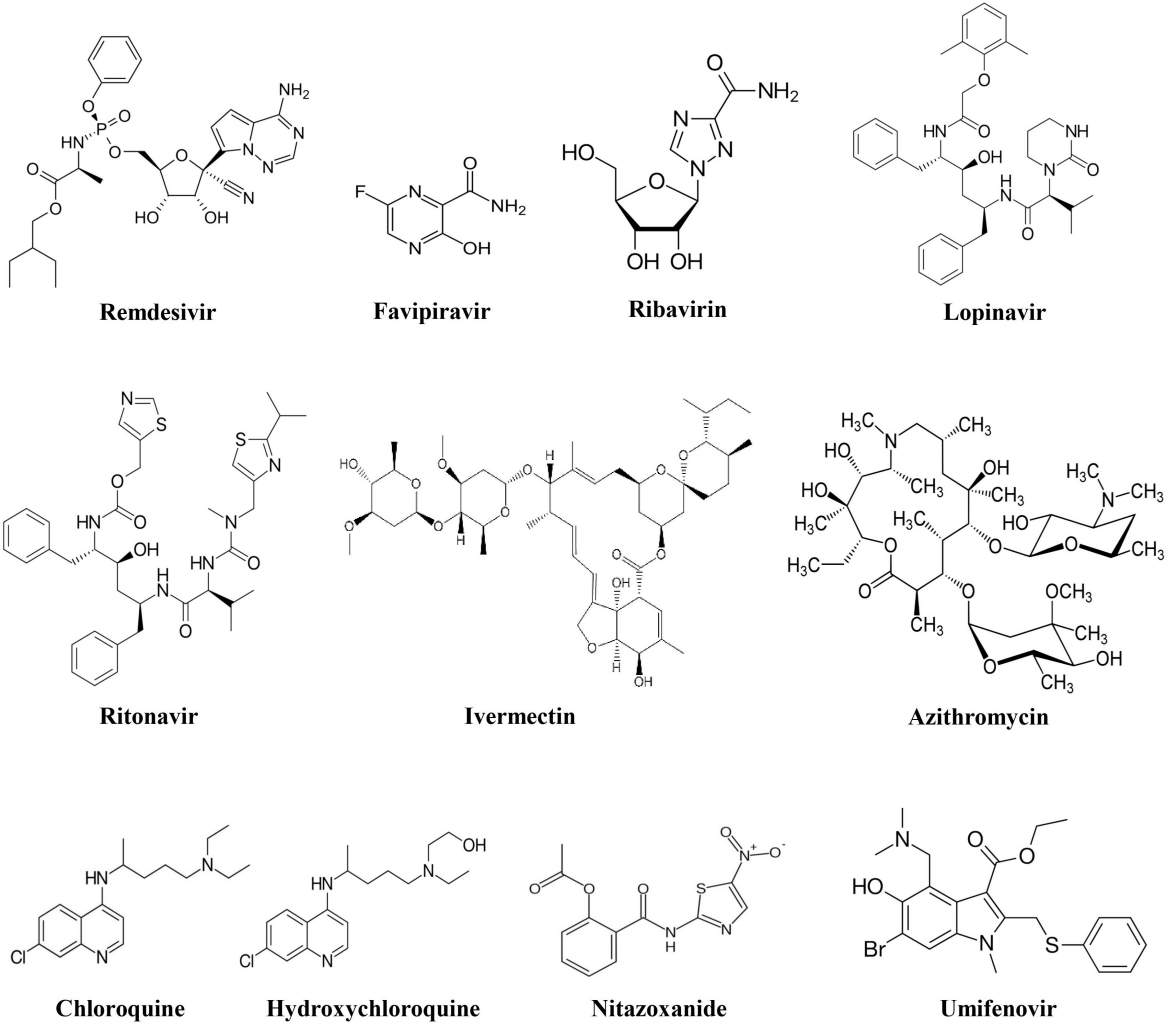
Chemical structures of potential investigational nCOVID-19 therapeutic agents.

**Figure 4:**
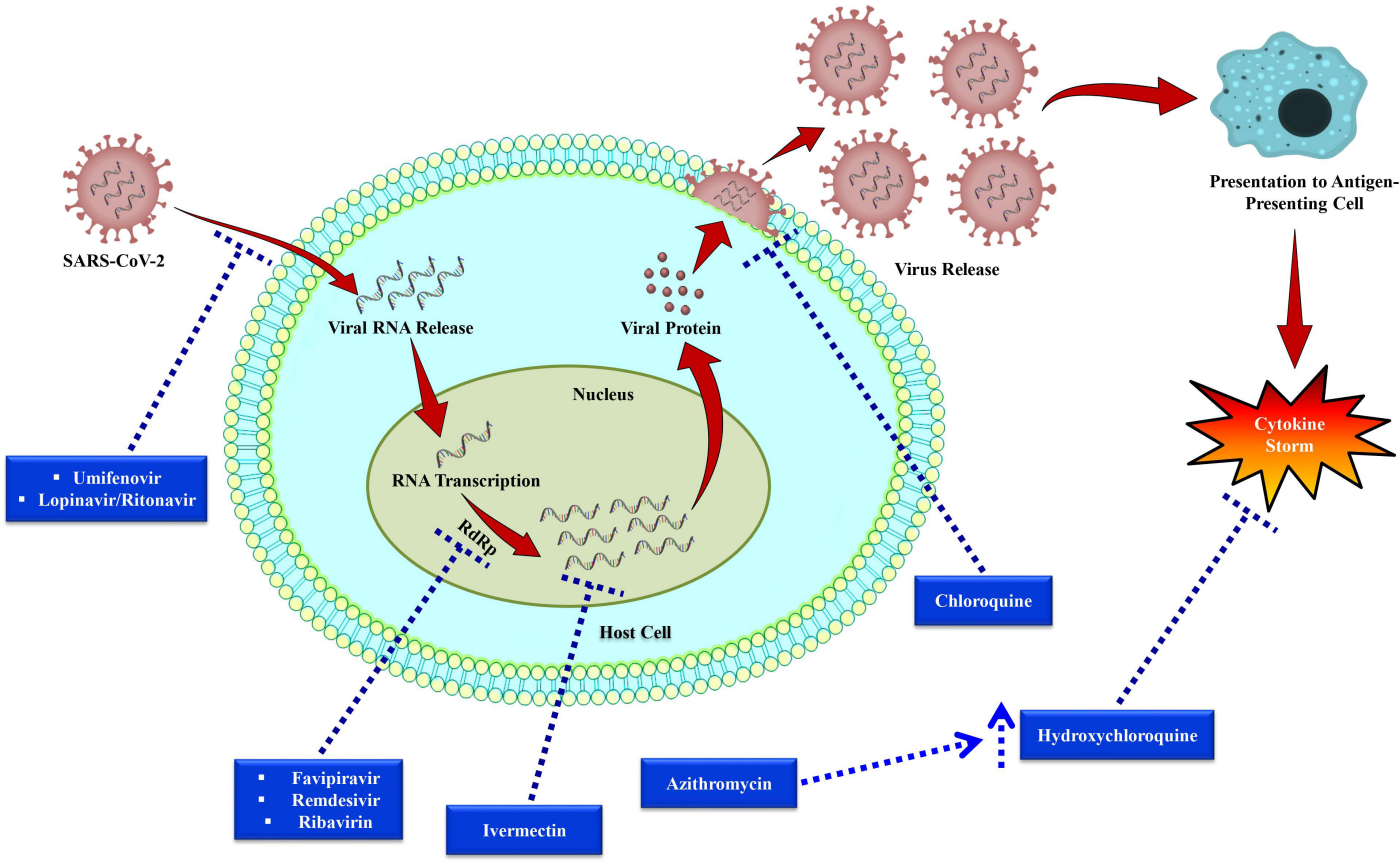
Replication cycle of SARS-CoV-2 and potential anti-SARS-CoV-2 drug targets. Following bindings of SARS-CoV-2 particles with the cell receptors including dipeptidyl peptidase 4 (DPP4), aminopeptidase N (APN), and angiotensin-converting enzyme 2 (ACE2), RNA of the virus then enters into the host cell and viral RNA transcription takes place via RNA-dependent RNA polymerase (RdRp). After that viral protein synthesis takes place that is encapsulated and then released from the host cell. Dotted arrows have been used to indicate the drug targets of investigational therapies for nCOVID-19.

Out of all the investigational drugs, remdesivir (Figure 3) has appeared as the most effective and promising antiviral drug (Li and De Clercq, 2020). This antiviral drug targets RNA-dependent RNA polymerase (RdRp) of the virus while escaping proofreading via viral exoribonuclease, (Agostini et al., 2018) which can ultimately lead to early termination of viral RNA transcription as given in Figure 4. Interestingly, remdesivir is a phosphoramidate prodrug and has a wide range of activities against numerous virus families, such as pneumoviridae, paramyxoviridae, filoviridae, and orthocoronavirinae (for example pathogenic MERS-CoV and SARS-CoV) (Sheahan et al., 2017; Martinez, 2020).

In a COVID-19 mouse model, when remdesivir was administered prophylactically and as early therapeutic intervention, it significantly decreased the pulmonary viral load, which ultimately reduced the progression of the disease and significantly improved respiration (Sheahan et al., 2017). In tissue culture models, Brown et al. (Brown et al., 2019) revealed that remdesivir showed half-maximal effective concentration (EC_50_) of 0.074 mM and 0.069 mM for MERS-CoV and SARS-CoV, successively (Brown et al., 2019). Furthermore, remdesivir (within the submicromolar EC50s) also effectively inhibited zoonotic CoV and human CoVs (HCoV-229E and HCoV-OC43) (Brown et al., 2019; Ko et al., 2020). Similar results were also observed when remdesivir was administered therapeutically (12 h post-inoculation) and prophylactically (24 h before prior inoculation) in MERS animal (rhesus macaque) model (de Wit et al., 2020). Even 2 amino acid substitutions (V553L and F476L) in the non-structural protein 12 polymerase were found to show lower-level of resistance toward remdesivir (Agostini et al., 2018).

In humans, pharmacokinetic data of remdesivir is not available. However, it has been revealed in rhesus monkeys that intravenous remdesivir administration at the dose of 10 mg/kg increased the intracellular concentration (over 10 mM) of active triphosphate form in peripheral blood mononuclear cells for a minimum of 24 h, (Warren et al., 2016) which is indicating its clinical significance in nCOVID-19 treatment. Furthermore, human safety data of remdesivir are available online (Mulangu et al., 2019). In USA, the first patient with confirmed nCOVID-19 was effectively treated with remdesivir for the advancement of pneumonia on 7th day of hospital admission in January, 2020 (Holshue et al., 2020). Moreover, to assess its efficacy and safety to treat individuals with confirmed nCOVID-19, phase III clinical trials (ClinicalTrials.gov, 2020e) have been started in March 2020. In that study, individuals received 200 mg of remdesivir on first day, subsequently received 100 mg/day. Although remdesivir showed promising *in vitro* and clinical activity against coronavirus (Sheahan et al., 2017; Holshue et al., 2020), recently it has been reported that there are some uncertainties because of its multiple adverse effects including hepatotoxicity, rectal hemorrhage (Jean et al., 2020b).

#### Favipiravir

In Japan, favipiravir (Figure 3) was primarily developed and approved as an anti-influenza drug (Shiraki and Daikoku, 2020; Wang et al., 2020b). This antiviral drug has a wide range of activities against various RNA viruses including rhinovirus, respiratory syncytial virus (RSV), and influenza. Former studies revealed that favipiravir was successfully used to treat infections associated with rabies, Lassa virus, and Ebola virus (Shiraki and Daikoku, 2020). Furthermore, favipiravir was also found to be effective to treat severe fever with thrombocytopenia syndrome (Shiraki and Daikoku, 2020). Nevertheless, favipiravir was found to be ineffective against DNA viruses.

Favipiravir is a potent antiviral drug that selectively suppresses the RdRp of RNA viruses (Figure 4) Favipiravir is likely to produce resistant viruses, as compared to oseltamivir (Shiraki and Daikoku, 2020). Indeed, this feature of favipiravir can be beneficial in the treatment of nCOVID-19. To treat influenza, favipiravir's recommended oral dose is 1,600 mg two times on first day, subsequently 600 mg twice/day from day 2 to 5, and 600 mg once/day on the sixth day. In recent times, initial findings of clinical trials have revealed that favipiravir exhibited significant activity in treating Chinese nCOVID-19 patients (Table 2) (Xinhua News Agency). In China, favipiravir has been approved to treat nCOVID-19 in March 2020. Furthermore, in China, randomized controlled trials involving nCOVID-19 patients are also assessing the efficacy of favipiravir plus baloxavir marboxil (an antiviral drug) (Qiu et al., 2020) and favipiravir plus IFN-α (Arab-Zozani et al., 2020).

**Table 2:** Potential investigational therapies for nCOVID-19.

**Drug class**	**Drug**	**Mechanism of action**	**Outcome**	**References**
Antiviral agents	Remdesivir	Inhibits viral RNA-dependent RNA polymerase (RdRp)	Showed promising *in vitro* and clinical activity against coronavirus	Brown et al., 2019; ClinicalTrials.gov, 2020e
	Favipiravir		Exhibited excellent activity in treating nCOVID-19 patients	Xinhua News Agency, 2020
	Ribavirin		It showed effective antiviral activity against SARS-CoV, but can be detrimental for the patients with respiratory distress	Chan et al., 2015; Martinez, 2020
	Umifenovir	Inhibits entry of virus into the host cell	It can inhibit viral entry into the host cell	Boriskin et al., 2008
	Lopinavir/ritonavir (LPV/r)		Combination of umifenovir and LPV/r showed better activity as compared to the sole use of LPV/r in nCOVID-19 treatment	Deng et al., 2020
Antimalarials	Chloroquine	Prevents the viral fusion with the cell membrane of the host cell	Findings from *in vitro* studies are promising	Vincent et al., 2005; Cortegiani et al., 2020; Wang et al., 2020b
	Hydroxychloroquine	Controls cytokine storm	Showed excellent *in vitro* activity as well as more potent and less likely to interact with other drugs as compared to chloroquine	Yao et al., 2020
Macrolide antibiotics	Azithromycin	Enhances the anti-SARS-CoV-2 effect of hydroxychloroquine	Combined use led to a reinforcement of hydroxychloroquine's efficacy in treating nCOVID-19 patients	Gautret et al., 2020
Glycopeptide antibiotics	Teicoplanin and its derivatives	Inhibits cathepsin L and cathepsin B in host cells	They can selectively suppress the effects of cathepsins B and L in the host cell	Wang et al., 2016; Zhou et al., 2016
Antiparasitic agent	Ivermectin	Dissociates IMPα/β1 heterodimer	Recent *in vivo* study has been demonstrated that it can remarkably decrease the level of viral RNA	Caly et al., 2020
	Nitazoxanide	Interferes with the host-regulated pathways linked with viral replication	Exerted a potent *in vitro* antiviral activity against SARS CoV-2	Wang et al., 2020b

#### Ribavirin

Ribavirin (Figure 3) is a RdRp inhibitor (Figure 4) used to treat various viral infections, for example, infections caused by RSV and hepatitis C virus (HCV) (Ogawa and Morisada, 2002). It was revealed by *in vitro* studies that when ribavirin was administered at a concentration of 50 mg/mL, it showed effective antiviral activity against SARS-CoV (Chan et al., 2015). Unfortunately, this antiviral drug was found to decrease the level of hemoglobin, therefore it can be detrimental for individuals with respiratory distress (Martinez, 2020).

### Viral Entry Inhibitors

#### Umifenovir

Umifenovir (Figure 3) is a potent antiviral agent that has a wide-range of activities against various viruses including HCV, influenza A and B viruses (Boriskin et al., 2008). Umifenovir's mechanism slightly varies with different viruses. It has been revealed that umifenovir suppresses the fusion of the virus with the host cell membrane (Figure 4), thus the subsequent viral entry into the host cell is inhibited (Boriskin et al., 2008).

#### Lopinavir/Ritonavir

In a clinical trial, it has recently been observed that lopinavir/ritonavir (LPV/r, Figure 3) protease inhibitors that are mainly used in human immunodeficiency virus (HIV) treatment did not significantly improve the nCOVID-19 symptoms (Cao et al., 2020). Furthermore, in a different study, effect of umifenovir plus LPV/r was compared with the sole treatment with LPV/r to treat nCOVID-19 (Deng et al., 2020). The findings of that study revealed that better effects were observed with the treatment of umifenovir plus LPV/r in comparison with the sole LPV/r treatment (Deng et al., 2020). However, more studies are required to evaluate the incidence of resistance and efficacy. As coronavirus becomes activated on the membrane of the host cell, thus combination of LPV/r and umifenovir are likely to inhibit/prevent the viral entry into the host cell (Figure 4). Besides, there is also a need regarding a better understanding of the more precise mechanisms to improve future clinical applications.

### Inhibitors of Viral Fusion and Cytokine Storm

#### Chloroquine

Chloroquine (Figure 3) is mainly used as an antimalarial drug. Furthermore, chloroquine is also used to treat various autoimmune disorders including rheumatoid arthritis and lupus erythematosus. In an animal model, it has recently been observed that chloroquine can also play a role as a potent antiviral drug against various viruses including influenza H5N1 (Yan et al., 2013). Interestingly, chloroquine can prevent the viral fusion with the cell membrane of host cell by increasing endosomal pH (Figure 4). Glycosylation of SARS-CoV's cellular receptors can also be interfered by chloroquine (Vincent et al., 2005; Wang et al., 2020b). Even though findings from *in vitro* studies regarding chloroquine is auspicious (EC_90_ = 6.90 mM, used nCOVID-19-infected Vero E6 cells), however use of chloroquine to treat nCOVID-19 infection is a completely off-label use. Furthermore, this drug is not strongly indicated due to some of its safety reasons including QT prolongation with ventricular dysrhythmia and adverse reactions on the renal, hepatic, and hematologic systems (Cortegiani et al., 2020).

#### Hydroxychloroquine

Hydroxychloroquine (a chloroquine derivative, Figure 3) is also mainly used as antimalarial and anti-inflammatory drugs (Sinha and Balayla, 2020). It has been proposed that hydroxychloroquine controls cytokine storm (Figure 4), which takes place in critically ill late phase nCOVID-19 patients (Yao et al., 2020). As compared to chloroquine, hydroxychloroquine is more potent and their EC_50_ values are 5.47 and 0.72, successively. In addition to this, hydroxychloroquine is less likely to interact with other drugs as compared to chloroquine. Moreover, in comparison with chloroquine phosphate, pharmacokinetic data confirmed that hydroxychloroquine is much more effective (5 days before) at inhibiting SARS-CoV-2 *in vitro* (Yao et al., 2020). It has been declared on March 26, 2020 by Taiwan CDC that hydroxychloroquine has a significant role in the treatment of nCOVID-19 patients. However, treatment with hydroxychloroquine is contraindicated for the patients who are pregnant or breastfeeding, allergic to hydroxychloroquine, glucose-6-phosphatase deficient, and for individuals with prolonged QT interval in electrocardiograms and retinopathy (Gautret et al., 2020).

### Enhancer of Anti-SARS-CoV-2 Effect of Hydroxychloroquine

#### Azithromycin

Previously, azithromycin (Figure 3) showed excellent *in vitro* activity against Ebola virus (Madrid et al., 2016). It was found that azithromycin was administered to individuals with viral infection, it prevented severe infections of respiratory tract in pre-school children (Bacharier et al., 2015). In a recent study, when azithromycin was administered (i.e., 500 mg on first day, then 250 mg per day from day 2-5), it remarkably reinforced the hydroxychloroquine's efficacy (when 200 mg was administered 3 times/day for 10 days) to treat 20 severely ill nCOVID-19 patients (Figure 4). The mean serum concentration of hydroxychloroquine was 0.46 ± 0.20 mg/mL. It is assumed that this excellent virus eliminating activity was achieved owing to the use of the aforesaid combination therapy (Gautret et al., 2020). Therefore, use of azithromycin along with hydroxychloroquine can be an effective future alternative to remdesivir in nCOVID-19 treatment. However, in this regard, a possible complication related to prolonged QT interval should be taken into consideration.

### Inhibitors of Cathepsins B and L

#### Teicoplanin and Its Derivatives

Teicoplanin is a glycopeptide antibiotic and it has been revealed by Zhou et al. (Zhou et al., 2016) that teicoplanin exerted inhibitory activity (IC_50_ as low as 330 nm) against replication- and transcription-competent virus-like particles. Studies confirmed that teicoplanin can suppress the entry of MERS and SARS envelope pseudotyped viruses (Wang et al., 2016; Zhou et al., 2016). In terms of its mechanism, teicoplanin can selectively suppress the effects of cathepsins B and L in host cell. These proteases are involved with cleaving the viral glycoprotein permitting exposure of the receptor-binding domain of its core genome and then release into the cytoplasm of host cells (Zhou et al., 2016; Baron et al., 2020). Therefore, teicoplanin blocked the entry of Ebola virus in the late endosomal pathway. Also, the derivatives of teicoplanin including telavancin, dalbavancin, and oritavancin, were also found to block the entry of SARS, MERS, and Ebola viruses (Zhou et al., 2016). Collectively, these findings suggest that teicoplanin and its derivatives might play a vital role in inhibiting the viruses that are dependent on cathepsin L (Table 2).

### Immunomodulators

#### Ivermectin

Ivermectin (Figure 3) is an antiparasitic agent and it has broad-spectrum of activity (Caly et al., 2020), Recent *in vitro* studies have revealed that this drug also has an antiviral effect against dengue and HIV viruses (Wagstaff et al., 2012). It has been found that the preformed IMPα/β1 heterodimer is accountable for the transport of viral protein into the nucleus and ivermectin can dissociate this heterodimer. Since this transport of viral protein into the nucleus is important for the replication cycle and suppression of the host's antiviral response, thus targeting this viral protein transport might prove as a significant target in the development of therapeutic agents against RNA viruses (Caly et al., 2012; Yang et al., 2020a). Following 48 h of nCOVID-19 infection, a recent *in vivo* study has been demonstrated that ivermectin can decrease the level of viral RNA (Figure 4) up to 5,000-times (Caly et al., 2020). Since ivermectin has an established safety profile as an antiparasitic agent, thus now it is needed to establish a safe and effective dose of this drug in clinical trials to treat nCOVID-19 infection.

#### Nitazoxanide

Nitazoxanide (Figure 3) is an effective antiparasitic and antiviral drug (Rossignol, 2016). This drug has a broad-spectrum *in vitro* antiviral activity against a range of viruses including RSV, rotavirus, parainfluenza, influenza, and coronavirus (Rossignol, 2016). In Vero-E6 cells, nitazoxanide exerted a potent *in vitro* antiviral activity against SARS CoV-2 (EC_50_ = 2.12 μM, at 48 h) (Wang et al., 2020b). Furthermore, this strong antiviral effect is in line with the observed EC_50_ values for nitazoxanide (EC_50_ = 0.92 μM) and tizoxanide (an active metabolite of nitazoxanide) (EC_50_ = 0.83 μM) against MERS-CoV in LLC-MK2 cells (Rossignol, 2016). In terms of its mechanism of action, it is believed that nitazoxanide has potent antiviral effect because of its ability to interfere with the host-regulated pathways associated with viral replication instead of the virus-specific pathways (Rossignol, 2016). Therefore, studies were carried out to evaluate the ability of this drug to treat influenza and other related acute respiratory infections. In the phase IIb/III of a clinical trial, positive effects of nitazoxanide were observed in the management of influenza symptoms, where 600 mg of nitazoxanide was orally administered twice a day (Haffizulla et al., 2014). Unfortunately, in phase II clinical trial it was observed that nitazoxanide neither alleviated the symptoms nor decrease the length of stay in hospitals of individuals infected with respiratory viruses (Gamiño-Arroyo et al., 2019). However, *in vitro* data regarding the activity of nitazoxanide against coronavirus is promising. Therefore, further studies are required to estimate its potential in nCOVID-19 treatment.

#### Janus Kinase Inhibitors

Baricitinib most commonly used in rheumatoid arthritis treatment. This drug is a reversible and selective inhibitor of Janus kinase 1 (JAK1) and JAK2. It has been found that these latter mentioned enzymes transduce intracellular signals for growth factors and cytokines associated with immune response, inflammation, and haematopoiesis. Moreover, this JAK inhibitor blocks the activities of AP2-associated with protein kinase 1, which ultimately prevents viral binding with the alveolar epithelium (Mayence and Vanden Eynde, 2019). It has also been indicated that baricitinib might be used as an additional therapy for the COVID-19 treatment (Richardson et al., 2020). In order to determine the safety and efficacy of sarilumab, hydroxychloroquine, lopinavir/ritonavir, and baricitinib to treat 1,000 hospitalized COVID-19 patients, a non-randomized phase II clinical study has recently been started (Scavone et al., 2020). Other selective JAK inhibitors including ruxolitinib, fedratinib, and sunitinib might also be effective against COVID-19 in decreasing endocytosis of virus, inflammation, and levels of cytokines including IL-6 and IFN-γ (Bekerman et al., 2017; ClinicalTrials.gov, 2020f; Favalli et al., 2020; Scavone et al., 2020; Stebbing et al., 2020).

### Convalescent Plasma

Previously, convalescent plasma therapy was used as a terminal therapy to increase the survival rate of individuals with a range of viral infections including SARS, severe infection caused by Ebola virus, pandemic 2009 influenza A H1N1, H5N1 avian influenza (Chen et al., 2020a; Shen et al., 2020). Convalescent plasma therapy can be effective because viremia can be suppressed due to the presence of plasma immunoglobulin antibodies in recovering patients. In a study, Shen et al. (2020) evaluated the effect of convalescent plasma therapy in 5 severely ill nCOVID-19 patients with ARDS. In that study, convalescent plasma was transfused in those patients with a novel coronavirus-specific antibody (neutralization titer >40 and binding titer > 1:1000). The used convalescent plasma of that study was obtained from five nCOVID-19-recovered individuals. The obtained convalescent plasma was then administered to the 5 patients (in between 10 and 22 days following admission) along with methylprednisolone and antiviral drugs. After convalescent plasma transfusion, clinical conditions of the patients were found to be improved, including decreased viral loads (patients became nCOVID-19 negative within 12 days), elevated level of SARS-CoV-2-specific enzyme-linked immunosorbent assay (ELISA), neutralizing antibody titers, normalized body temperature (within 3 days in four/five patients), improved ARDS (four patients at 12 days following transfusion), successful weaning from mechanical ventilation (three participating individuals within 2 weeks of therapy), increased partial pressure of oxygen/fraction of inspired oxygen, and reduced score in sequential organ failure assessment. Out of the 5 participants, 2 of them were in stable condition (at 37 days following transfusions), while 3 of them were discharged from the hospital (following 51, 53, and 55 days of staying in the hospital) (Shen et al., 2020). Finally, the researchers summarized that although there were a small number of participants in this study, they suggested the therapy with convalescent plasma can be effective in the nCOVID-19 treatment (Shen et al., 2020).

### Monoclonal or Polyclonal Antibodies and Other Potential Therapies

As a prophylactic measure and therapy, monoclonal and polyclonal antibodies (targeting hemagglutinin binding) have been recommended to treat various viral infections including influenza (Beigel et al., 2019). The effectiveness of these antibodies against MERS-CoV largely encouraged the recent efforts to develop monoclonal and polyclonal antibodies against coronaviruses (Sheahan et al., 2017). For instance, in a phase I trial, SAB-301 (a human polyclonal antibody) which was produced in transchromosomic cattle was found to be safe and better tolerated in healthy participants (Beigel et al., 2019). In a study, Cockrell et al. (2016) revealed in mouse models that human monoclonal antibodies (mAbs)-based immunotherapy only mediated protection in the early stage of MERS (Martinez, 2020).

Many *in vitro* analyses showed that S protein of SARS-CoV is crucial to mediate the viral entry into the host cells. In addition to this, the cleavage and subsequent activation of the S protein of SARS-CoV via a host cell's protease is vital for the entry of the virus (Glowacka et al., 2011). In cell cultures, it has been noticed that transmembrane serine protease 2 (TMPRSS2) is a vital protease of host cells that causes activation of S protein of SARS-CoV, therefore it was studied as an important target for antiviral drugs (Sheahan et al., 2017). Previously, camostat mesylate (an inhibitor of serine protease) showed inhibitory activity against TMPRSS2 (Kawase et al., 2012). Furthermore, K11777 (a cysteine protease inhibitor) exhibited significant inhibitory activity (at submicromolar range) against replication of MERS-CoV and SARS-CoV (Zhou et al., 2015). Sarilumab is a human monoclonal antibody and 3 clinical trials are ongoing to assess the safety and efficacy of this antibody (alone or along with other standard therapies) in nearly 1,500 COVID-19 patients (ClinicalTrials.gov, 2020a,d,f; Scavone et al., 2020).

Eculizumab (a monoclonal antibody) is approved to treat neuromyelitis spectrum disorders, refractory generalized myasthenia gravis, and atypical hemolytic uraemic syndrome. This monoclonal antibody inhibits the terminal portion of the inflammatory response-associated complement cascade. Although the function of the complement cascade in nCOVID-19 pathogenesis is not clear, numerous studies revealed that its suppression may effectively function as a therapeutic technique (Ip et al., 2005; Yuan et al., 2005; Gralinski et al., 2018). Due to these findings, eculizumab will be tested in the SOLID-C19 clinical trial to treat individuals with severe ARDS and nCOVID-19 (ClinicalTrials.gov, 2020b). Currently, emapalumab (a monoclonal antibody) is being studied in an open-label, randomized, phase II/III study to evaluate the safety and efficacy of this antibody in decreasing respiratory distress and hyper-inflammation in nCOVID-19 patients (ClinicalTrials.gov, 2020c).

In China, stem cells are currently being studied as a treatment for nCOVID-19. Tocilizumab (a mAb) is an immunosuppressive agent and is used to treat rheumatoid arthritis (Kaneko, 2013). This agent was designed to suppress the IL-6 binding with its receptors to alleviate cytokine storm syndrome. Tocilizumab is now being studied as a potential nCOVID-19 treatment (Jean et al., 2020b; Slater, 2020).

### Herbal Medicines

In nCOVID-19 high-risk populations, traditional Chinese medicines were also regarded as a preventative measure, based on the traditional uses and anecdotal evidence of prevention of H1N1 pdm09 and SARS. Nonetheless, there is a lacking of clinical data regarding the effectiveness of these herbal medicines as an nCOVID-19 treatment (Cunningham et al., 2020; Luo et al., 2020). In China, several traditional medicines were widely used during the nCOVID-19 epidemic and 6 of these herbal medicines include Lianqiao (*Fructus forsythia*), Jinyinhua (Lonicerae Japonicae Flos), Gancao (Glycyrrhizae Radix Et Rhizoma), Baizhu (Atractylodis Macrocephalae Rhizoma, rhizome of *Atractylodes macrocephala* Koidz), Fangfeng (Saposhnikoviae Radix, dried root from the perennial herb *Saposhnikovia divaricate*), and Huangqi (Astragali Radix, dried root of *Astragalus membranaceus Bge*. *Var*. *mongholicus*). Indeed, stringent clinical studies are required with a large number of participants to demonstrate the preventive role of these traditional Chinese medicines (Cunningham et al., 2020; Luo et al., 2020).

### Adjunctive Medications

#### Antimicrobial Agents

The occurrence of co-infection can widely vary among the patients with confirmed nCOVID-19. Various reports suggest that several co-pathogens including viruses (such as rhinovirus, influenza, and HIV) and bacteria (for example *Candida* species, *Mycoplasma pneumonia*) can co-exist in these patients. Among them, influenza A virus was most commonly found to co-exist (Jean et al., 2020b). Furthermore, nCOVID-19 patients with pneumonia were found to be commonly treated by the coadministration of anti-influenza drugs and antibiotics (Jean et al., 2020b). Therefore, careful selection of potential broad-spectrum antibiotic(s) is required for the long-stay (over 6 days) hospitalized patients (Chou et al., 2019; Jean et al., 2020a).

#### Corticosteroids

Mixed clinical findings were observed with the use of corticosteroids to treat SARS-CoV infections. Although various reports suggested that there was no significant contribution of corticosteroids in clinical outcomes (Stockman et al., 2006). In contrast, it was suggested by a report that decreased mortality rate was observed due to the use of corticosteroids in critically ill patients (Chen et al., 2006a; Wu et al., 2020a). Unfortunately, several reports suggested worse outcomes including longer time for viral clearance, or elevated composite endpoint of ICU admission or even death, owing to the use of corticosteroids (Auyeung et al., 2005). In a cohort (*n* = 309), a longer time in viral clearance was observed in the corticosteroids-receiving MERS-CoV patients (Arabi et al., 2018). Nevertheless, in the same study, it was observed that there was an insignificant decrease in 90-day mortality in corticosteroids-receiving patients. Recent reports suggested that there was a decreased rate of mortality in nCOVID-19 patients with ARDS due to the use of corticosteroids (Wu et al., 2020a).

These findings suggest that use of corticosteroids resulted in inconsistent outcomes. However, corticosteroids might be beneficial for patients with cytokine-linked lung injury and those who might rapidly develop progressive pneumonia (Shang et al., 2020). Indeed, healthcare professionals need to carefully assess the risk and benefit ratio of corticosteroid use for each patient. This necessity to assess risk and benefit of corticosteroid use in individual patients and its careful dose consideration has been demonstrated in diagnosis and treatment guidelines from China's National Health Commission. As per that guideline, glucocorticoid (equivalent to methylprednisolone 1-2 mg/kg per day for three-five days or less) may be considered based on chest imaging and respiratory distress. Large-dose of glucocorticoids can suppress the immune system, this can result in delayed SARS-CoV-2 clearance (McCreary and Pogue, 2020). Recently, Chinese Thoracic Society recommended a lower dose of methylprednisolone (≤ 0.5–1 mg/kg per day) for a maximum of 7 days in selected patients, prior to treatment these selected patients should be carefully assessed for potential risks and benefits (Shang et al., 2020). More clinical studies are immediately required to elucidate the function of corticosteroids in nCOVID-19.

#### Angiotensin II Receptor Blockers, ACE Inhibitors, and Statins

In a study, Yang et al. (2020b) mentioned that diabetes and cerebrovascular diseases were the commonly observed comorbidities in the non-survivors of nCOVID-19 in ICUs. Furthermore, Guan et al. (2020) also observed similar results in their study and these nCOVID-19 patients received angiotensin II receptor blockers (ARBs) or ACE inhibitors. Indeed, SARS-CoV-2 and SARS-CoV can bind with the ACE2 receptors on the epithelial cells of lung, kidney, and intestine (Fang et al., 2020). Therefore, when ARDS is not present, ARB or ACE inhibitors can be administered to nCOVID-19 patients. Increased activity of ACE2 was found to be linked with decreased severity of ARDS among individuals with RSV-caused lower respiratory tract infection (Wösten-van Asperen et al., 2013). Interestingly, Fedson (2016) revealed in their study that statins mainly target host response to infection, instead of the virus itself. These researchers also indicated that combination therapy with statins and ARB may induce the reversal of homeostatic processes, which will allow the self-recovery of individuals (Fedson et al., 2020).

#### Non-steroidal Anti-inflammatory Drugs

There is an argument regarding the usage of non-steroidal anti-inflammatory drugs (NSAIDs) like ibuprofen since it can increase the ACE2 receptors (Day, 2020). If the severely ill nCOVID-19 individuals suffer from fever, acetaminophen can be a good option to control body temperature as compared to other NSAIDs (Therapeutics Initiative, 2020).

#### Anticoagulant Therapy

Tang et al. (2020) confirmed that anticoagulant therapy by heparin (an anticoagulant) specially with low molecular weight heparin improved the prognosis in severely ill patients with nCOVID-19. Furthermore, 28-day mortality of heparin receivers was found to be lower as compared to the non-users among individuals with sepsis-stimulated coagulopathy scores 4 or D-dimer > 6-times the upper limit of normal (Tang et al., 2020).

#### Enhancing Immunity by Vitamins and Minerals in nCOVID-19

##### Vitamin A

In human body, vitamin A plays various important functions including protecting mucosal and epithelium integrity, mediating growth and development, and proper maintenance of vision (Huang et al., 2018). Vitamin A is also essential for enhancing immune response and maintaining regulatory action in both humoral and cellular immune responses (Huang et al., 2018). In case of infants, supplementation with vitamin A was found to ameliorate antibody response following several vaccines including anti-rabies (Siddiqui et al., 2001) and measles vaccination (Huang et al., 2018). Moreover, an improved immune response to influenza virus vaccination has also been reported in children (2–8 years) who had a deficiency of vitamin A and D at baseline, following supplementation with vitamin A and D (Patel et al., 2019).

##### Vitamin D

Vitamin D has a significant contribution in modifying both adaptive and innate immune responses (Aranow, 2011). It has been revealed by epidemiological studies that there is a link between deficiency of vitamin D and elevated susceptibility to acute viral respiratory infections (Monlezun et al., 2015). It has also been suggested that vitamin D significantly modulates the innate immune responses against various viral respiratory infections including RSV, parainfluenza 1 and 2, and influenza A and B (Zdrenghea et al., 2017). Indeed, studies have revealed that there is a strong relationship between vitamin D deficiency and elevated risk of both lower and upper respiratory tract infections (Jolliffe et al., 2013). Nonetheless, conflicting and heterogeneity in dosage regimens and baseline vitamin D conditions in study populations were observed in randomized controlled trials (RCTs) (Jolliffe et al., 2013). In a study, Aglipay et al. (2017) observed no significant difference between the action of high-dose (2000 IU per day) vs. standard-dose (400 IU per day) vitamin D supplementation on viral upper respiratory tract infections (Aglipay et al., 2017). Nevertheless, only one-third of the study subjects received vitamin D at doses below 30 ng/ml. Vitamin D increased the plasma level of TGFβ without ameliorating antibody generation in a RCT on the effect of vitamin D administration on influenza vaccine response in deficient elderly person (Goncalves-Mendes et al., 2019). In addition to this, it was also indicated in the latter mentioned RCT that vitamin D administration perhaps directed the polarization of lymphocyte toward a tolerogenic immune response (Goncalves-Mendes et al., 2019). In a different RCT, monthly administration of high-dose of vitamin D (100,000 IU/month) decreased the occurrence of acute respiratory infections in older long-term care residents as compared to a standard dose group (12,000 IU/month) (Ginde et al., 2017). Therefore, it is quite clear that the effect of vitamin D on antiviral immunity against respiratory infections is dependent on an individual's vitamin D status. Moreover, it has been confirmed that vitamin D supplementation is also useful in case of other viral infections, for instance, vitamin D addition to conventional Peg-α-2b/ribavirin therapy for treatment-naive individuals with chronic HCV genotype 1 infection considerably ameliorated the viral response (Abu-Mouch et al., 2011), and similar action was also seen in individuals with HCV genotype 2–3 (Nimer and Mouch, 2012).

##### Vitamin E

Vitamin E possesses strong antioxidant property and it can modify host immune responses [14]. The deficiency of this vitamin can lead to impairment of both cellular and humoral immune responses (Moriguchi and Muraga, 2000). Some studies revealed that administration of vitamin E may exert harmful activities in case of infectious disease. Vitamin E increased the risk of pneumonia among 50–69 years old adult smokers (Hemilä and Kaprio, 2008). Similarly, vitamin E supplementation (200 IU/day) did not significantly reduce the respiratory tract infections in elderly nursing facility residents (Meydani et al., 2004). Nevertheless, in a small pilot RCT, positive activities of vitamin E were seen in the treatment of chronic hepatitis B, where vitamin E administration markedly normalized the liver enzymes and HBV-DNA negativization (Andreone et al., 2001). Similarly, in a RCT, vitamin E supplementation increased anti-HBe seroconversion and virological response in the pediatric population (Fiorino et al., 2017).

##### Vitamin C

Vitamin C plays a significant role as an enzymatic cofactor in numerous physiological reactions including immune potentiation, collagen synthesis, and hormone generation (Kim et al., 2013). In mouse models, it was revealed that vitamin C plays important role in the antiviral immune responses against the influenza A virus (H3N2) via the elevated generation of IFN-α/β, particularly at the early stages of infection (Kim et al., 2013). Nonetheless, no significant benefit has been observed in using mega-dose of vitamin C as a prophylactic measure to lower the incidence of common cold caused by viral infections (Hemilä and Chalker, 2013).

##### Zinc

Zinc (an essential trace element) contributes significantly in the growth, development, and maintenance of immune responses (Prasad, 2013; Read et al., 2019). The deficiency of zinc is linked with an enhanced susceptibility toward infectious diseases, for example, viral infections. An individual's zinc status is a vital factor that can affect the immune response against viral infections. Indeed, zinc-deficient individuals are at greater risk of developing infections including HCV or HIV (Read et al., 2019). Acevedo-Murillo et al. (2019) reported that there was a noticeable clinical improvement in the 103 children (1 month−5 years) with pneumonia in the zinc-receiving group as compared to placebo (Acevedo-Murillo et al., 2019). The researchers also confirmed that there was a rise in the cytokine response in Th1 pattern (INF-γ and IL-2) only in the zinc-receiving group, along with Th2 cytokines (IL-10 and IL-4) being increased or remained elevated in both groups. Following stem cell transplantation, oral administration of a high dose of zinc (150 mg/day) increased thymic activity and output of new CD4^+^ naive T cells, which eventually helped in the prevention of Torque Teno virus reactivation (Iovino et al., 2018). Nonetheless, Provinciali et al. (1998) summarized that prolonged administration of zinc (400 mg/day) or zinc plus arginine (4 d/day) in the elderly (age 64–100 years) people restored zinc concentrations in plasma, which was ineffective in stimulating or improving the antibody response or number of CD3, CD4, or CD8 lymphocytes following influenza vaccination.

##### Selenium

Selenium (a trace element) also exerts a range of important functions including antioxidant effects, various pleiotropic activities, and anti-inflammatory effects (Rayman, 2012). Selenium deficiency is found to be linked with cognitive impairment, poor immune response, and elevated risk of mortality, whereas an increased level of selenium or treatment with selenium has exhibited antiviral actions (Rayman, 2012). Broome et al. (2004) assessed whether an increased selenium administration (50–100 μg/day) ameliorated immune response in adults with a borderline concentration of selenium (Broome et al., 2004). Treatment with selenium elevated the plasma selenium levels, and also increased the activities of cytosolic glutathione peroxidase and lymphocyte phospholipid. Furthermore, selenium also increased the cellular immune responses (elevated level of IFN-γ and other cytokines), along with an increased level of T-helper cells and earlier peak T-cell proliferation. Nonetheless, it was observed that humoral immune responses were not affected (Broome et al., 2004). Moreover, selenium treatment in participants also induced rapid poliovirus clearance.

##### Copper

Copper (another essential trace element) has a significant contribution in the differentiation and development of immune cells (Li et al., 2019). It has also been confirmed that copper exerted *in vitro* antiviral effects. Intracellular copper was found to regulate the life cycle of influenza virus (Rupp et al., 2017), while thujaplicin-copper chelates inhibited the replication of human influenza viruses (Miyamoto et al., 1998). In a study, Turnlund et al. (2004) determined the effects of chronic administration of copper on immune response, oxidative stress, and indices of copper status. These researchers observed that when copper was administered at a dose of 7.8 mg/day, copper significantly increased the level of superoxide dismutase, benzylamine oxidase, and plasma ceruloplasmin activity as compared to 1.6 mg/day dose, which further suggesting an enhancement in antioxidant status. Nonetheless, increased copper administration (7.8 mg/day) markedly decreased the proportion of antibody titer, serum IL-2R, and circulating neutrophils against the Beijing strain of influenza (Turnlund et al., 2004).

##### Magnesium

Magnesium (an essential mineral) has a significant contribution in regulating immune response via significantly affecting the T helper-B cell adherence, macrophage response to lymphokines, Immunoglobulin M (IgM) lymphocyte binding, adherence with immune cells, antibody-dependent cytolysis, and immunoglobulin synthesis (Liang et al., 2012). It has also been reported in *in vivo* and *in-vitro* studies that magnesium may have a contribution in the immune function against viral infections (Chaigne-Delalande et al., 2013).

## Controversies Regarding nCOVID-19 Treatments

Still now there is no specific antiviral drug to treat nCOVID-19, but some of the investigational drugs were found to be useful. Various drugs are being analyzed *in vitro* studies or clinical trials. Although ribavirin is a potent antiviral drug, its clinical effects are not clear and its side effects ought to be carefully considered. On the other hand, chloroquine has been studied in 15 interventional studies. Furthermore, in the Chinese Clinical Trial Registry, the derivatives of chloroquine were prospectively registered; and more studies are required to evaluate their antiviral effects and to estimate the recommended dose in nCOVID-19 patients (Zhang et al., 2020a). Along with antiviral drugs, glucocorticoids ought to be utilized carefully and in a timely manner in nCOVID-19 patients. In addition to this, extracorporeal support need to be considered under strict contraindications and indications, otherwise, there will be numerous additional complications and also a waste of resources (Zhang et al., 2020a).

## Future Research Directions

In order to manage the current nCOVID-19 outbreak, extensive measures are needed to be taken to lower the person-to-person transmission of the virus. In addition to this, special efforts and attention are required to reduce or protect the susceptible populations such as elderly people, health care providers, and children. More studies are also essential to understand the mechanisms related to nCOVID-19 pathogenesis. This better understanding will help the development of specific and effective therapies against SARS-CoV-2. Since the respiratory tract is mainly affected by SARS-CoV-2, thus special consideration is required to deliver the drug into the respiratory tract. More studies in animals and clinical trials on drug repositioning can also be considered to identify potential drugs to treat nCOVID-19.

## Conclusion

Still there is no available specific drug or vaccine to treat nCOVID-19, thus effective preventative measures are recommended. Specific drugs are urgently required to inhibit the entry of the virus and subsequent replication to overcome this outbreak. Currently, as mentioned in this article, multiple investigational drugs and clinical trials are ongoing. The discovery of new drugs will ultimately enable us to better control this outbreak. Furthermore, *in silico* studies can also be considered to faster the drug development process. Finally, sharing findings or data will be effective to fight against nCOVID-19 globally.

## Author Contributions

MK and MU conceived the original idea and designed the outlines of the study and prepared the figures for the manuscript. MK wrote the initial draft of the manuscript. MU revised and improved the draft. MH, JA, MA, GA, SB, MB-J, MA-D, and LA participated in the literature review of the manuscript. All authors have read and approved the final manuscript.

## Conflict of Interest

The authors declare that the research was conducted in the absence of any commercial or financial relationships that could be construed as a potential conflict of interest.

## Abbreviations

ACE2: angiotensin converting enzyme 2
AP: antigen presentation
APCs: antigen presentation cells
APN, aminopeptidase N, ARBs: angiotensin II receptor blockers
ARDS: acute respiratory distress syndrome
CDC: Centers for Disease Control
nCOVID-19: novel coronavirus disease 2019
CoVs: coronaviruses
DPP4: dipeptidyl peptidase 4
dsRNA: double-strand RNA
EC_50_: half maximal effective concentration
ED: emergency department
ELISA: enzyme-linked immunosorbent assay
EUA: emergency use authorization
FDA: Food and Drug Administration
GGO: ground-glass opacity
HCV: hepatitis C virus
HIV, human immunodeficiency virus;, MHC: major histocompatibility complex
or HLA: human leukocyte antigen
ICU: intensive care unit
IL-6: interleukin 6
LPV/r: lopinavir/ritonavir
mAbs: monoclonal antibodies
MERS: Middle East respiratory syndrome
N7-MTase: N7-methyltransferase
NSAIDs: nonsteroidal anti-inflammatory drugs
PRRs: pattern recognition receptors
PUI: patient under investigation
RdRp: RNA-dependent RNA polymerase
RSV: respiratory syncytial virus
S protein: spike protein
SAM: S-adenosyl-methionine
SARS: severe acute respiratory syndrome
SARS-CoV-2: Severe acute respiratory syndrome coronavirus 2
TMPRSS2: transmembrane serine protease 2
WHO: World Health Organization.
